# Dextran Sodium Sulphate-Induced Gastrointestinal Injury Further Aggravates the Impact of Galantamine on the Gastric Myoelectric Activity in Experimental Pigs

**DOI:** 10.3390/ph14060590

**Published:** 2021-06-18

**Authors:** Jan Bures, Ilja Tacheci, Jaroslav Kvetina, Vera Radochova, Darina Kohoutova, Martin Valis, Stanislav Rejchrt, Veronika Knoblochova, Jana Zdarova Karasova

**Affiliations:** 1 2nd Department of Internal Medicine-Gastroenterology, Charles University, Faculty of Medicine in Hradec Kralove and University Hospital, 500 03 Hradec Kralove, Czech Republic; tacheci@gmail.com (I.T.); kvetina.jaroslav@seznam.cz (J.K.); darina.kohoutova@rmh.nhs.uk (D.K.); rejchrt@lfhk.cuni.cz (S.R.); veronika.knoblochova@fnhk.cz (V.K.); 2 Animal Laboratory, Faculty of Military Health Sciences, University of Defence, 500 01 Hradec Kralove, Czech Republic; vera.radochova@unob.cz; 3 The Royal Marsden NHS Foundation Trust, London SW3 6JJ, UK; 4 Department of Neurology, Charles University, Faculty of Medicine in Hradec Kralove and University Hospital, 500 03 Hradec Kralove, Czech Republic; martin.valis@fnhk.cz; 5 Department of Toxicology and Military Pharmacy, Faculty of Military Health Sciences, University of Defence, 500 01 Hradec Kralove, Czech Republic; zdarova.jana@gmail.com; 6 Biomedical Research Centre, University Hospital, 500 05 Hradec Kralove, Czech Republic

**Keywords:** Alzheimer disease, galantamine, electrogastrography, experimental pigs, gastric motor dysmotility, small bowel transit time, wireless capsule enteroscopy

## Abstract

Galantamine has been used as a treatment for Alzheimer disease. It has a unique, dual mode of action (inhibitor of acetylcholinesterase and allosteric modulator of nicotinic acetylcholine receptors). Nausea (in about 20%), vomiting (10%) and diarrhoea (5–7%) are the most common side effects. The aim of this study was to assess the effect of galantamine on porcine gastric myoelectric activity without (Group A) and with (Group B) dextran sodium sulphate (DSS)-induced gastrointestinal injury. Galantamine hydrobromide was administrated to twelve pigs as a single intragastric dose (24 mg). Gastric myoelectric activity was investigated by electrogastrography (EGG). Basal (15 min before galantamine administration) and study recordings after galantamine administration (300 min) were evaluated using a running spectral analysis. Results were expressed as dominant frequency of gastric slow waves and power analysis (areas of amplitudes). Altogether, 3780 one-minute EGG recordings were evaluated. In Group A, power was steady from basal values for 180 min, then gradually decreased till 270 min (*p* = 0.007). In Group B, there was a rapid gradual fall from basal values to those after 120 min (*p* = 0.007) till 300 min (*p* ˂ 0.001). In conclusion, galantamine alone revealed an unfavourable effect on porcine myoelectric activity assessed by gastric power. It can be a plausible explanation of galantamine-associated dyspepsia in humans. DSS caused further profound decrease of EGG power. That may indicate that underlying inflammatory, ischaemic or NSAIDs-induced condition of the intestine in humans can have aggravated the effect of galantamine on gastric myoelectric activity.

## 1. Introduction

Galantamine has been used as a treatment for Alzheimer disease and myasthenia gravis [1,2,3,4,5]. It has a unique, dual mode of action, it works as a reversible competitive inhibitor of acetylcholinesterase and a modulator of nicotinic acetylcholine receptors (type I positive allosteric modulator of α7nACh receptors). Galantamine inhibits the breakdown of acetylcholine by binding to the active site on acetylcholinesterase. This inhibitory effect is particularly important in the cerebral areas with the most affected cholinergic neurotransmission in Alzheimer disease (i.e., frontal cortex and hippocampus). Peak serum concentration after its oral administration is reached in one hour in humans, half-life elimination lasts about 7 h [1,2,3,4,5]. Further, galantamine acts as a weak competitive reversible acetylcholinesterase inhibitor in the gastrointestinal tract [6]. Galantamine can also influence the immune system through a “cholinergic anti-inflammatory pathway [5].

Nausea (occurring in about 20% of chronic users), vomiting (10%) and diarrhoea (5–7%) are the most common gastrointestinal adverse side effects [7,8,9,10,11,12,13,14]. Pathogenesis of these motility disorders has not been fully clarified yet. Ageing has a significant impact on the function of the gastrointestinal tract, even in healthy seniors. Its function can be further deteriorated by an underlying inflammatory or ischemic condition of the intestine. Experimental dextran sodium sulphate (DSS)-induced injury affects morphology and/or function of different parts of the gastrointestinal tract, including the small bowel [15,16,17,18,19,20,21,22,23,24,25], and thus, interfere with the absorption and pharmacokinetics of different drugs. In experimental setting, DSS can also induce functional gastric motor disorder [26].

Surface electrogastrography (EGG) is a non-invasive method for the assessment of gastric myoelectrical activity [27,28,29,30,31,32]. Our group has demonstrated that EGG is also reliable in experimental pigs [26,33]. Porcine surface EGG is fully comparable with that one recorded in healthy humans [34]. EGG has also been used for the evaluation of gastric motility influenced by inhibitors and modulators of acetylcholinesterase in experimental pigs [35,36].

The aim of this study was to assess the effect of a single dose of galantamine on porcine gastric myoelectric activity with and without DSS-induced injury.

## 2. Results

**Figure 1:**
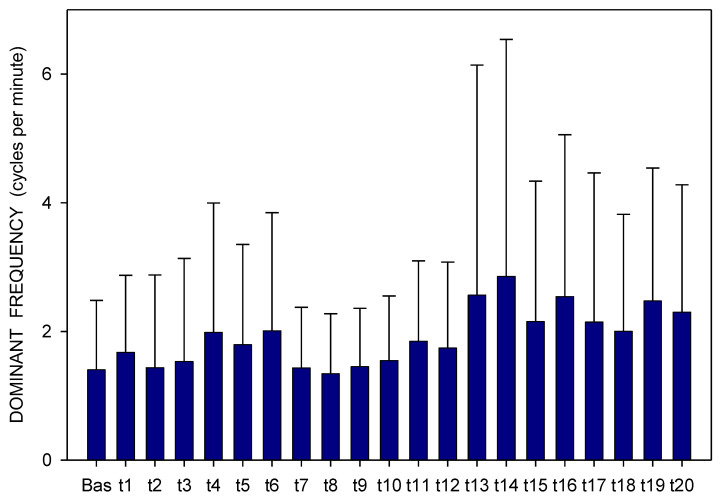
Electrogastrography. Dominant frequency before and after a single intragastric dose of 24 mg galantamine (mean + standard deviation). Explanatory note: Bas: 15-min basal recording before galantamine administration; tn: 15-min study recordings after galantamine administration (t1: time interval between 0–15 min ... t20: time interval between 286–300 min).

**Figure 2:**
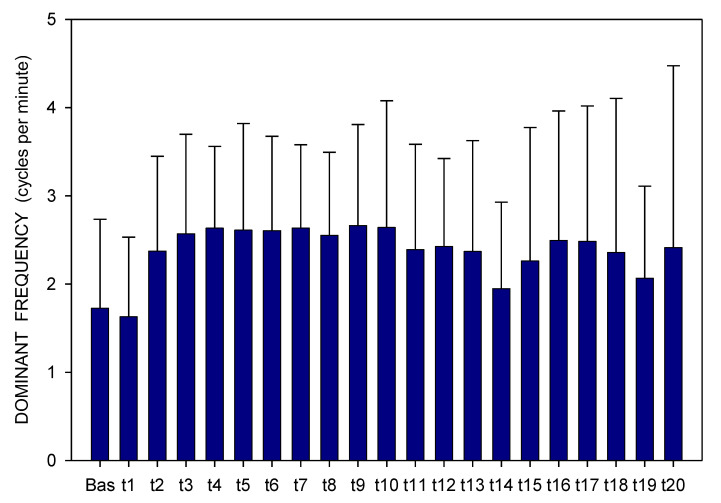
Electrogastrography. Dominant frequency before and after a single intragastric dose of 24 mg galantamine in animals with previous 7-day administration of dextran sodium sulphate (mean + standard deviation). Explanatory note: Bas: 15-min basal recording before galantamine administration; t: 15-min study recordings after galantamine administration (t1: time interval between 0–15 min ... t20: time interval between 286–300 min).

**Figure 3:**
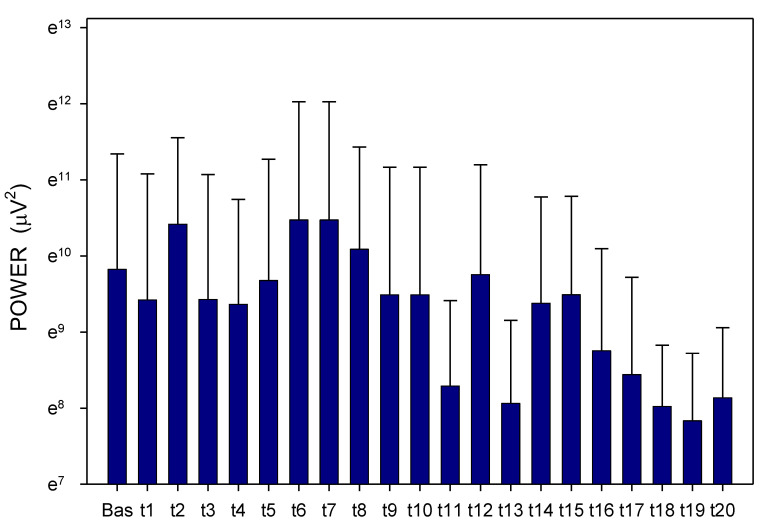
Electrogastrography. EGG power before and after a single intragastric administration of 24 mg galantamine (mean + standard deviation). Outliers omitted. Axis Y: natural logarithm scale. Explanatory note: Bas: 15-min basal recording before galantamine administration; t: 15-min study recordings after galantamine administration (t1: time interval between 0–15 min ... t20: time interval between 286–300 min).

**Figure 4:**
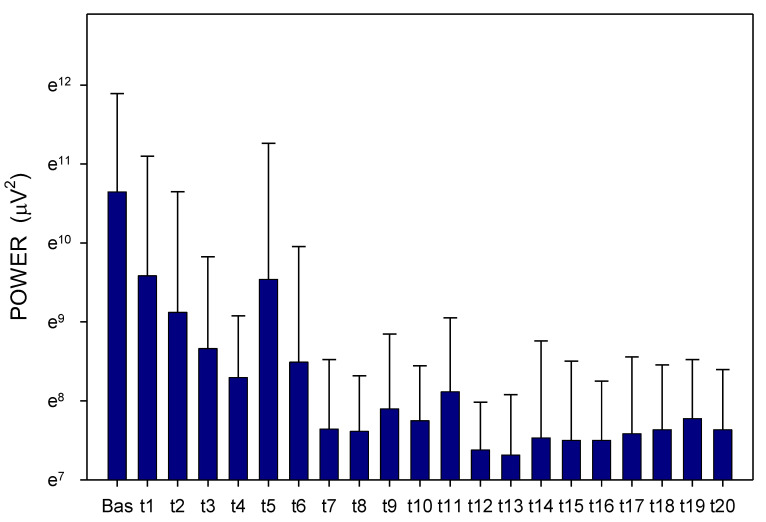
Electrogastrography. EGG power before and after a single intragastric administration of 24 mg galantamine in animals with previous 7-day administration of dextran sodium sulphate (mean + standard deviation). Outliers omitted. Axis Y: natural logarithm scale. Explanatory note: Bas: 15-min basal recording before galantamine administration; t: 15-min study recordings after galantamine administration (t1: time interval between 0–15 min ... t20: time interval between 286–300 min).

Altogether, 3780 one-minute EGG recordings were evaluated, each in dominant frequency (DF) and power. A total of 41 outliers (1.1% of all recordings; from various time intervals of different animals in both groups) were excluded from the final evaluation of the EGG power. In Group A, DF raised gradually from basal values (median 1.06; interquartile range 0.70–1.41 cpm) significantly after 60 min (1.17; 0.94–1.41 cpm; *p* = 0.016) and 210 min (1.41; 0.94–2.81 cpm; *p* ˂ 0.001), Figure 1. In Group B, DF increased from basal values (1.17; 0.94–2.81 cpm) rapidly within 30 min (2.58; 1.29–3.28 cpm; *p* ˂ 0.001) and sustained significantly higher till the end of recording (2.11; 1.17–3.3 cpm; *p* ˂ 0.001), Figure 2. In Group A, power was steady from basal values (median 2264; IQR 901–7743 μV^2^) for 180 min, then gradually decreased till 270 min (1461; 558–3393 μV^2^; *p* = 0.007), Figure 3. In Group B, there was a rapid gradual fall from basal values (1919; 690–5646 μV^2^) to those after 120 min (1297; 648–535 μV^2^; *p* = 0.007) till 300 min (1216; 315–2740 μV^2^; *p* ˂ 0.001), Figure 4. Basal values of DF were significantly lower in Group A compared to Group B (*p* = 0.004). DF was lower in Group A during the whole study period, the most distinct differences between the groups were identified after 30 min (*p* < 0.001) and from 105 to 120 min after the administration of galantamine (*p* < 0.001), see Figure 1 and Figure 2. Basal values of the EGG power of both groups were not significantly different (*p* = 0.336; power 0.365), see Figure 3 and Figure 4. No gross gastrointestinal pathology was revealed on autopsy in any animal tested.

**Figure 5:**
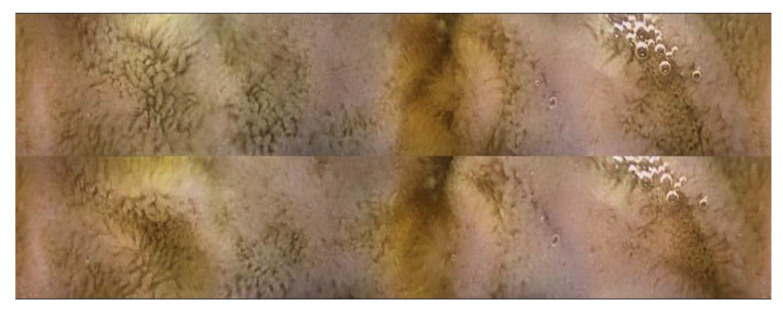
Wireless capsule enteroscopy. Normal jejunum.

**Figure 6:**
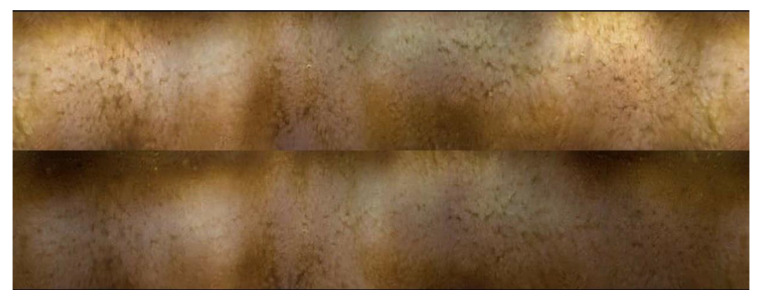
Wireless capsule enteroscopy. Normal ileum.

Wireless capsule enteroscopy enabled successful investigation of the entire small bowel in all animals. All endoscopic findings were normal in all pigs (Figure 5 and Figure 6). Small bowel transit time of particular animals were: 205, 322, 592, 288, 421 and 445 min (mean 379 ± 137; median 371.5).

**Figure 7:**
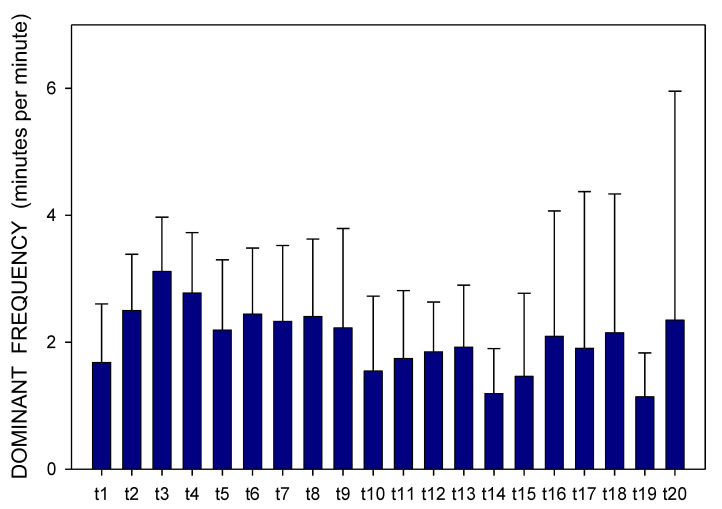
Electrogastrography. Dominant frequency in animals with a shorter small bowel transit time (272 ± 60 min); mean + standard deviation. Explanatory note: t: 15-min study recordings after galantamine administration (t1: time interval between 0–15 min ... t20: time interval between 286–300 min).

**Figure 8:**
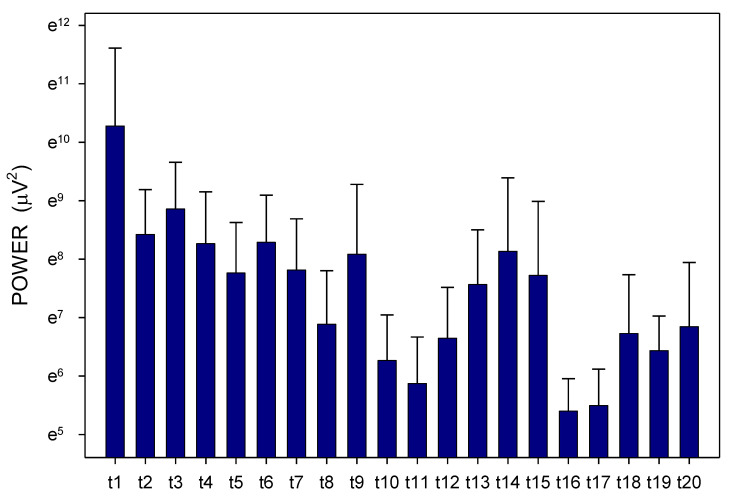
Electrogastrography. EGG power in animals with a shorter small bowel transit time (272 ± 60 min); mean + standard deviation. Outliers omitted. Axis Y: natural logarithm scale. Explanatory note: t: 15-min study recordings after galantamine administration (t1: time interval between 0–15 min ... t20: time interval between 286–300 min).

**Figure 9:**
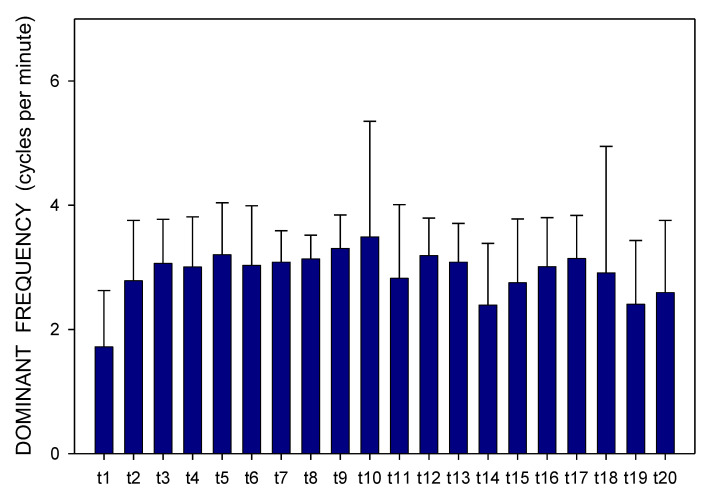
Electrogastrography. Dominant frequency in animals with a longer small bowel transit time (486 ± 93 min); mean + standard deviation. Explanatory note: t: 15-min study recordings after galantamine administration (t1: time interval between 0–15 min ... t20: time interval between 286–300 min).

**Figure 10:**
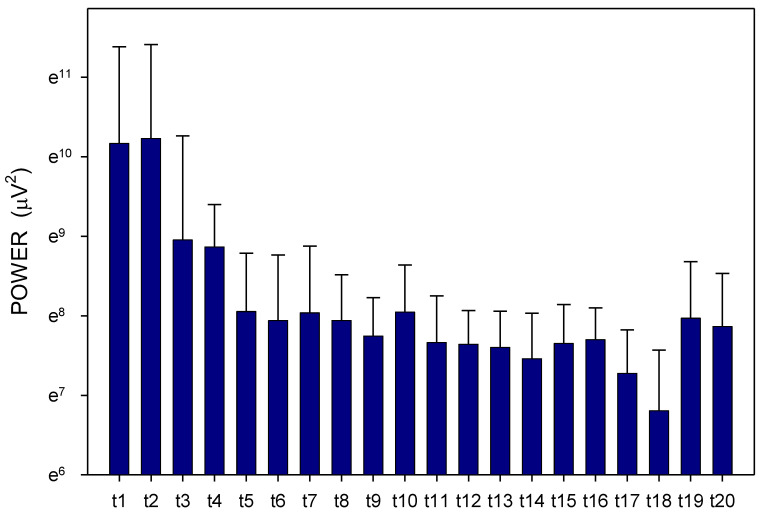
Electrogastrography. EGG power in animals with a longer small bowel transit time (486 ± 93 min); mean + standard deviation. Outliers omitted. Axis Y: natural logarithm scale. Explanatory note: t: 15-min study recordings after galantamine administration (t1: time interval between 0–15 min ... t20: time interval between 286–300 min).

Animals were split according to small bowel transit time into subgroups of shorter (3 pigs; 272 ± 60 min) and longer transit time (3 pigs; 486 ± 93 min). Dominant frequency and power were assessed in these two subgroups separately (Figure 7, Figure 8, Figure 9 and Figure 10). There was a clear trend towards lower values of dominant frequency in pigs with a shorter transit time. Most prominent significant differences were revealed in time intervals t10 (median 1.17 vs. 3.52; *p* < 0.001), t14 (median 0.94 vs. 3.05; *p* < 0.001), t19 (median 0.94 vs. 2.81; *p* < 0.001) and t20 (median 0.94 vs. 2.81 cycles per minute; *p* < 0.001). There were significant differences of the EGG power in animals with a shorter transit time compared to pigs with a longer transit time. Most prominent differences were found in time intervals t2 (median 1804 vs. 4313; *p* = 0.003), t11 (median 236 vs. 2037; *p* < 0.001) and t16 (median 188 vs. 2226 μV^2^; *p* < 0.001).

## 3. Discussion

Our current study brought completely novel findings on the impact of galantamine on gastric motor function in experimental pigs. Porcine gastrointestinal physiology is similar to the human one [37,38], therefore, preclinical experimental studies may be relevant for clinical medicine. To the best of our knowledge, this is the first study evaluating effect of galantamine on the gastric motor function in experimental pigs. The impact of DSS on the entire gastrointestinal tract depends on three variables: molecular weight of DSS, daily dose and cumulative dose of DSS. Different doses of medium-molecular-weight DSS have been recommended (from 0.25 to 1.25 g/kg/day) for induction of experimental gastrointestinal injury [15,16,17,18,19,20,21,22,23,24,25,26,39]. We intentionally decided on administration the lower dose (0.3 g/kg/day) to induce functional but not a structural gastrointestinal injury. Basal and study values of DF were significantly higher in the group with previous DSS administration compared to the group without DSS. However, it is necessary to interpret these findings with caution as mean values of DF remained mostly within normal range between 1.5 and 3 cycles per minute.

Porcine gastric motor activity has a substantial inter-individual variability, even in experimental animals without any intervention [our data not shown]. That is why the basal recording serves as an individual control for subsequent study part in each experimental pig. We measured small bowel transit time in particular animals. We divided pigs into two subgroups, with a shorter and longer transit time and evaluated dominant frequency and power. Animals with a shorter transit time had lower values of dominant frequency and EGG power. The porcine small intestine is about 12 m long (twice as long compared to the human small bowel). Therefore, a sufficient reserve of an intestinal absorptive capacity can be assumed. However, small bowel transit time has an important impact on drug absorption and pharmacokinetics. In our previous study with donepezil, experimental pigs with a shorter bowel transit time had a significantly lower average plasma concentrations of donepezil compared to animals with a longer small bowel transit time [40].

It can be assumed, that the impact of galantamine on the porcine EGG power could be dose-dependent—in analogy how the cholinergic and anti-cholinergic compounds act. In our previous study, we found that high doses of atropine (0.15 mg/kg) induce an important decrease of EGG power in experimental pigs in contrast to moderate dose of atropine (0.05 mg/kg) [34]. Parkman et al. [41] studied EGG before and after a low dose of atropine in humans (0.6 mg as an intravenous bolus continued by 0.25 mg per hour i.v.). Atropine caused a decrease of EGG power under fasting condition [41]. Cardiac response to atropine in humans also depends on the dose. Atropine in a standard dose (0.5–1 mg) increases the heart rate in healthy subjects. However, atropine can cause paradoxical heart rate slowdown when given in low doses (i.e., <0.5 mg)—presumably as a result of the interactions in the central nervous system. One of the proposed mechanisms for the paradoxical bradycardia effect of atropine at low doses involves the blockade of the inhibitory presynaptic muscarinic receptors, thereby blocking a system that inhibits the parasympathetic response [42]. In our current experimental study, the course of EGG power in the second group (galantamine + DSS) was similar to that which was observed after the intramuscular administration of neostigmine in experimental pigs in a standard dose (0.015 mg/kg) [43]. Neostigmine is a parasympathomimetic compound that acts as a reversible acetylcholinesterase inhibitor. By interfering with the breakdown of acetylcholine, neostigmine indirectly stimulates both nicotinic and muscarinic receptors [43].

Turiiski et al. [44] studied the impact of galantamine on gastrointestinal motility in rats. They found several functional disturbances: hypertonia (caused by tonic contractions in smooth muscles), increased gastric and ileal peristalsis and accelerated intestinal transit time. These reactions were dose-dependent (concentrations of galantamine from 10^−7^ mol/L to 10^−4^ mol/L). The tonic and phasic effect of galantamine was partially reversed by atropine or ipratropium bromide [44].

Galantamine may cause malignant ventricular arrhythmias (due to prolongation of QT interval and by influencing potassium channels in cardiac myocytes) [45]. We have not noticed such an event in any animal tested.

We are aware of possible limits of our study. Our current study was performed in female pigs only. Sex hormones can influence porcine gastrointestinal motor activity. In our previous experimental study on oesophageal manometry we found differences between male and female experimental pigs [46]. Our trial was designed as an acute study with higher but a single dose of galantamine. Chronic administration of galantamine with or without DSS can bring further important findings.

## 4. Materials and Methods

### 4.1. Animals

Twelve experimental adult female pigs (*Sus scrofa* f. *domestica*, hybrids of Czech White and Landrace breeds; 3-month-old; mean weight 33.2 ± 1.9 kg) were enrolled into the study. The animals were purchased from a certified breeder (Stepanek, Dolni Redice, Czech Republic; SHR MUHO 2050/2008/41). The pigs were housed in an accredited vivarium (Faculty of Military Health Sciences, Hradec Kralove, Czech Republic). All animals were fed with a standard assorted A1 food (Ryhos, Novy Rychnov, Czech Republic) with equal amounts twice a day and had free access to a drinking water.

### 4.2. Design of the Study

Experimental pigs were randomly divided into two groups (six animals in Group A, six animals in Group B). All procedures were carried out under general anaesthesia. Intramuscular injections of ketamine (20 mg per kg; Narkamon, Spofa, Praha, Czech Republic) and azaperone (2.2 mg per kg; Stresnil, Janssen Animal Health, Saunderton, UK) were used as an induction of the anaesthesia in all animals. Intravenous infusion of propofol (AstraZeneca AB, Stockholm, Sweden) was used for subsequent maintenance of general anaesthesia. Heart rate and pulse oximetry were monitored throughout the experiments.

DSS was administered to overnight fasting animals in a dietary bolus in the morning at 7 a.m. for 7 days (10 g per day) to 6 out of 12 animals (Group B). Galantamine hydrobromide was administrated in the morning at 7 a.m. as a single intragastric dose (Group A: 24 mg) without previous DSS and after previous administration of DSS (Group B; 24 mg of galantamine). The whole dose of galantamine was administrated endoscopically, using a video-gastroscope GIF-Q180 (Olympus Optical Co, Tokyo, Japan) dedicated for animal use only. Dextran sodium sulphate salt was purchased from Sigma-Aldrich (Praha, Czech Republic). Galantamine hydrobromide was purchased from Mylan Pharmaceuticals (Praha, Czech Republic).

**Figure 11:**
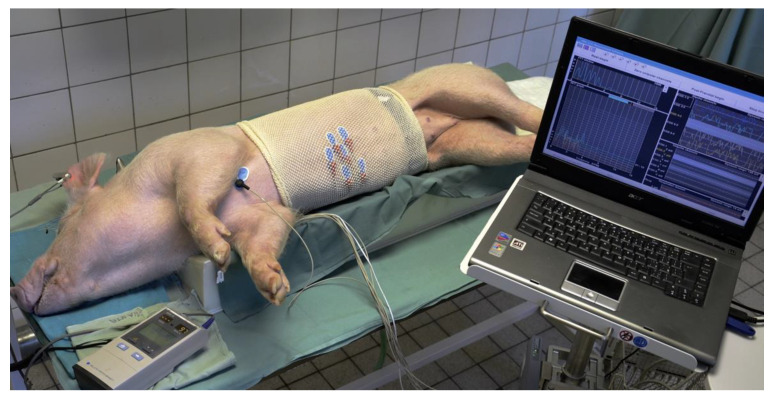
Arrangement of electrogastrography in experimental pigs. All recordings were accomplished under general propofol anaesthesia. Monitoring of vital functions was performed throughout the procedures.

Our original method of porcine surface EGG was published already [33]. We used six active self-adhesive electrodes placed on the upper part of the abdomen, the 7th basal electrode was placed to the left of the middle sternum. A special abdominal belt enabled to identify artefacts caused by breathing and body movements (Figure 11).

EGG recording was accomplished by means of the EGG Stand (MMS, Enschede, the Netherlands). Basal recording (within 15 min before galantamine administration) and study recordings after galantamine administration (throughout 300 min) were evaluated by an MMS software (version 8.19). Running spectral analysis was used for a standard evaluation of EGG. Results were conveyed as dominant frequency of gastric slow waves (DF; cycles per minute—cpm) and power analysis (areas of amplitudes; μV^2^).

Wireless capsule enteroscopy was performed on Day 8 in six animals of Group B. The investigation was performed by means of a CapsoCam system (CapsoVision, Saratoga, USA) which combines four camera images into a seamless 360° panoramic view of the small bowel. Capsules were introduced endoscopically into the duodenum by means of a special delivery system (AdvanCE, US Endoscopy, Mentor, OH, USA). After the examination had been completed, endoscopic capsules were captured and images were evaluated by a dedicated software. Capsule enteroscopy was also used for the assessment of small bowel transit time (time between the first acquired endoscopic image of the duodenum and the last image of the ileo-caecal valve) [40].

### 4.3. Statistical Analysis

Data was statistically treated by means of descriptive statistics and Mann–Whitney rank sum test using the SigmaStat software (Version 3.1, Jandel Corp, Erkrath, Germany).

### 4.4. Ethics

The Project was approved by the Institutional Review Board of the Animal Care Committee of the University of Defence (Protocol Number MO 171673/2019-684800), Faculty of Military Health Sciences, Hradec Kralove, Czech Republic. The study was conducted in accordance with the policy for experimental and clinical studies [47]. Animals were held and treated in conformity with the European Convention for the Protection of Vertebrate Animals [48].

## 5. Conclusions

Galantamine alone revealed an unfavourable effect on porcine myoelectric activity assessed by gastric power. It can be a plausible explanation of galantamine-associated dyspepsia in humans. The decline in power was more prominent in the group with DSS pre-treatment. That may indicate that an underlying disease and/or a drug-induced gastrointestinal injury in elderly humans can aggravated the effect of galantamine on gastric myoelectric activity.

## Data Availability

Availability of data and materials: all data generated or analysed during this study are included in this article.
